# Characterization of volatile flavor compounds from fish maw soaked in five different seasonings

**DOI:** 10.1016/j.fochx.2023.100805

**Published:** 2023-07-23

**Authors:** Xiaoqing Miao, Shuang Li, Shan Shang, Na Sun, Xiuping Dong, Pengfei Jiang

**Affiliations:** a School of Food Science and Technology, Dalian Polytechnic University, Liaoning, Dalian 116034, China; b National Engineering Research Center of Seafood, Liaoning, Dalian 116034, China

**Keywords:** Fish maw, Seasonings, Soaking, Flavor change, HS-GC-IMS, Volatile compounds

## Abstract

In this study, sensory evaluation, electronic nose, and HS-GC-IMS were used to investigate the effects of different seasonings (deionized water, onion, ginger, Sichuan pepper, and mixed seasonings) on the flavor of fish maw. The results showed that the volatile compounds of fish maw soaked in different seasonings were mainly organic sulfides and aromatic compounds. A total of 95 volatile compounds were identified, including 25 aldehydes, 23 olefins, 19 alcohols, 11 esters, 9 ketones, 3 acids, 2 sulfides, 1 furan, 1 ether and 1 ketoxime. Sichuan pepper group and mixed seasoning group had the most significant changes in volatile components, and had the most effective improvement on the flavor of fish maw compared with other groups. These findings will provide reference for producing high quality fish maw and improving its flavor quality. These findings will provide feasible theoretical support for the pretreatment and exploration of fish maw products in the future.

## Highlights

• The flavor of fish maw soaked in five different seasonings was studied.
• Organic sulfide and aromatic compound are the main components of seasoned fish maw.
• E-nose and HS-GC-IMS can distinguish five kinds of seasoned fish maw.
• The mixed seasoning group can effectively improve the flavor of fish maw.
• The characteristics of volatile components in seasoned fish maw were visualized.

## 1 Introduction

Fish maw, the dried swim bladders, is filled with gas and is the organ that fish use to maintain balance and sink and float. Fish maw has been promoted and consumed in Asia as a traditional delicacy and premium tonic (Clarke, 2002). Especially in China and Southeast Asia, fish maw has been used as a luxury tonic due to its multiple medicinal properties (such as promoting postpartum recovery and healing of cutaneous wounds; enhancing immunity and memory; and removing free radicals) (Dai et al., 2020, Howaili et al., 2020, Jian and Wu, 2003, Yu et al., 2009, Zhao et al., 2016). The demand and value of fish maw have risen with the development of society and are expected to continue to grow in the future.

Fish maw is usually prepared into soup or other dishes, and is favored for its soft texture and rich nutrition (Sadovy de Mitcheson et al., 2019; Sinthusamran & Benjakul, 2015). Flavor is one of the most important indicators to measure food quality. Volatile compounds in food have a decisive influence on the flavor of food. However, there has been little scientific reporting on the flavor of fish maw compared to other seafood products. Dried fish maws are rehydrated by soaking before eating, which is the preparation work required before cooking (Clarke, 2004, Newman, 2004). Due to the high flavor threshold of volatile compounds in fish maw, seasoning should be added to optimize the flavor during processing. In order to elucidate the flavor changes of fish maw during initial processing, we studied the changes of volatile compounds in fish maws soaked in water containing different seasoning (Li, Dong, Jiang, Qi, & Lin, 2022).

In the study of food flavor, headspace solid–gas chromatography-ion mobility spectrometer (HS-GC-IMS) has been widely used in the analysis of VCs in food. HS-GC-IMS is a technique for the rapid detection of volatile components in samples using GC combined with IMS (Xu, et al., 2023), which combines the high separation of gas chromatography and the high sensitivity of ion mobility spectrometry compared to the mainstream technique (GC–MS) used to study volatile flavor components (Chen et al., 2020). GC-IMS has been widely used for the detection of volatile flavor compounds in the field of food analysis (Li et al., 2020). Previously, our group also applied HS-GC-IMS technology to carry out a series of studies on food flavor (Chen et al., 2021; Jiang et al., 2022; Jiang et al., 2022; Jin et al., 2023).

In this study, HS-GC-IMS combined with electronic nose technology were applied to clarify the evolution and formation of volatile compounds in fish maw during soaking with different seasoning (deionized water, onion, gingerroot, Sichuan pepper, mixed seasonings), and the flavor fingerprints of fish maw were also established. Furthermore, principal component analysis (PCA) and heat map analysis were used to distinguish the characteristic compounds among different treated samples. This study aimed to provide theoretical evidence to control flavor and quality changes of fish maw during soaking. The research results will provide reference for producing high-quality fish maw and improving the flavor quality of fish maw during processing.

## 2 Material and methods

### 2.1 Material

Dried fish maws (*Gadus morhua*) were purchased from Beijing Tong Ren Tang Health (Dalian) Seafoods Co., Ltd. Onion, ginger, Sichuan pepper purchased from the local markets. Firstly, the cleaned fish maw was boiled in a water bath at 100 °C for 8 min, and then the fish maw was divided into 6 equal portions (500 g each) and soaked in different seasoning groups (A-F) for 24 h. A: control group (the boiled fish maw was not further treated); B: deionized water group (the boiled fish maw was soaked in deionized water (3000 mL)); C: onion group (onion (300 g) was boiled in deionized water (3000 mL) for 60 min, and cooled to obtain onion seasoning water); D: ginger group (ginger (300 g) was boiled in deionized water (3000 mL) for 60 min, and cooled to obtain ginger seasoning water); E: Sichuan pepper group (Sichuan pepper (75 g) was boiled in deionized water (3000 mL) for 60 min, and cooled to obtain Sichuan pepper seasoning water); F: mixed seasonings group (onion (300 g), ginger (300 g) and Sichuan pepper (75 g) were boiled in deionized water (3000 mL) for 60 min to obtain mixed seasoning water). The soaked fish maws were then used for subsequent experiments immediately.

### 2.2 Sensory evaluation

A total of 12 panelists (8 females and 4 males, aged from 19 to 30) ranked the fishy smell of boiled and soaked fish maws. In order to ensure the homogeneity of the samples tested by the panelists, the samples were divided into 10 equal parts, and the samples were divided into equal parts after mixing. After packaging, the samples were marked with 3 random numbers, and the samples were handed over to panelists for sensory evaluation in orthogonal Latin order. The evaluations were conducted in the evaluation compartment of the sensory laboratory, and the evaluators were not allowed to communicate with each other. Sensory evaluators observed, smelled, and tasted the samples according to the given sample number sequence, and then ranked the products according to a certain characteristic of the products. Sensory evaluators need to clear their mouths with water and chew unsalted soda crackers during the tasting interval (30 s) to remove the residual taste (Zhang, Ayed, Wang, & Liu, 2019). The evaluators ranked the samples according to the fishy smell, the fishiest ranked 1, and the least fishy ranked 6. Statistical analysis was carried out on the sorting results by Friedman test to realize the significance analysis of differences between samples. Multiple comparisons with the least significant difference value. The sensory data were collected and calculated by the online sensory analysis system SE, and the comparison value R between the two samples was obtained.

### 2.3 Electronic nose (E-nose)

The volatile compounds of the six group of boiled and soaked fish maws were determined using an electronic nose (PEN3.0, AIRSENSE, Germany). Boiled and soaked fish maw sample (1 g) was placed in a glass injection bottle and then equilibrated at 25 °C for 15 min. The detection parameters were set as follows: sample preparation time (5 s), detection time (60 s), auto -zero time of 10 s, and sensor cleaning time (60 s) and period (54–56 s) (Ma et al., 2021, Weng et al., 2021).

The main sensitive odors of each sensor were R1 (aromatic components), R2 (nitrogen oxides), *R*3 (ammonia and aromatic components), R4 (hydrides), R5 (short-chain alkanes), R6 (methyl groups), R7 (inorganic sulfides), R8 (alcohols, aldehydes, ketones), R9 (organic sulfides and aromatic compounds), and R10 (long-chain alkanes).

### 2.4 Gas chromatography-ion mobility spectrometry (GC-IMS)

HS-GC-IMS (FlavourSpec ®, Dortmund, Germany) was used to detect the volatile compounds of the soaked fish maw (Jin et al., 2021). Firstly, the soaked fish maw (2 g) was put into a headspace bottle (20 mL). After incubation (40 °C, 15 min), the headspace gas (500 μL) was driven into the column (MXT-5, 15 m, 0.53 mmID, 1.0 μm df, Restek Corporation, America) by the autosampler (80 °C) with high-purity nitrogen (99.999%) according to the set program (2 mL/min, 2 min; 10 mL/min, 8 min; 100 mL/min 10 min; 150 mL/min, 10 min; 60 °C). After GC separation, the analyte was ionized in the IMS ionization chamber (positive ion mode). Finally, the volatile compounds were identified by comparing the retention index (RI) and drift time (DT).

### 2.5 Statistical analysis

All experiments were performed at least in three independent trials. The data were analyzed by SPSS 18.0 software (SPSS Inc., Chicago, IL, USA) and statistical significance of differences with *p* < 0.05 was evaluated with LSD test. According to all volatile compounds identified between samples, a plug-in PCA score and biplot diagram (through normalization and eigenvectors) by the HS-GC-IMS instrument were also acquired.

## 3 Results and discussion

### 3.1 Analysis of sensory evaluation results

#### 3.1.1 Rank sum test statistical sorting results

The addition of seasonings can effectively improve the overall flavor of meat products (Huang et al., 2020). The results of least significant difference (LSD) analysis were shown in Table 1, The ranking results of the rank-sum test for fish maw flavor were shown in Table 2. According to Table 1 and Table 2, the score of fish maw soaked in different seasoning was significantly different. Among them, the mixed seasoning group had the best adsorption effect on the flavor of fish maw, indicating that onion, ginger, and Sichuan pepper in the mixed seasoning group had synergistic effect on the overall odor of fish maw, which could not only cover up the unpleasant odor, but also enhance the overall flavor of fish maw. The second highest score was in the Sichuan pepper group, which was rich in terpenes, flavonoids, and amides, and could reduce the unpleasant odor in fish maw through sensory coverage (Chen et al., 2021; Yang et al., 2021). Terpenoids, shogaol, and gingerols were found to be closely related to the pungent and aromatic flavor of ginger (Krüger, Bergin, & Morlock, 2018). Onion's unique flavor characteristics are mainly due to its widespread sulfur compounds (Wang, Luca, & Edelenbos, 2019). The sulfide in onion and terpene in ginger also had a masking effect on the flavor of fish maw. The results showed that the unique flavor compounds of the seasoning had significant effects on the overall flavor of fish maw samples.

**Table 1:** LSD analysis of flavor adsorption effect of fish maw.

Sample	A	B	C	D	E	F
A	N	N	Y	Y	Y	Y
B	N	N	N	Y	Y	Y
C	Y	N	N	Y	Y	Y
D	Y	Y	Y	N	Y	Y
E	Y	Y	Y	Y	N	N
F	Y	Y	Y	Y	N	N

Note: N means there is no significant difference between samples, Y means there is a significant difference between samples (*p* < 0.05). A: unseasoned group, B: deionized water group, C: onion group, D: ginger group, E: Sichuan pepper group, F: mixed seasonings group.

**Table 2:** Sorting results of flavor adsorption effect of fish maw.

Sample Average-rank	A	B	C	D	E	F
Average-rank	1.76^a^	1.80^b^	2.80^c^	3.96^d^	5.28^e^	5.40^f^

Note: Different lowercase letters in the same line represent the significant test results of LSD. The same letter means no significant difference between samples, while different letters mean significant difference between samples (*p* < 0.05). A, B, C, D, E, and F are the same as Table 1.

The ginger group also had a better adsorption effect on fish maw flavor, mainly because ginger contained a variety of olefin compounds. Among them, β-sesquiterpene was a characteristic flavor compound in ginger (Peng et al., 2022), which had a strong volatile aroma and spicy smell, and had a certain stimulating effect on the senses, so it had adsorption effect on fish maw flavor. The adsorption effect of the deionized water group on the flavor of fish maw was small, and the effect of the unseasoned group was the worst. The main reason was that volatile compounds in the fish maw partially dissolve into the deionized water, which changed the flavor of the fish maw. The unseasoned group did not play the role of sensory masking on fish maw, so it had no significant effect on its flavor.

#### 3.1.2 Statistical sorting method by R-index method

R-index is a method used to calculate the area of the subjects' perception curve, and the discrimination probability between paired samples represents the degree of difference between them (Feng et al., 2012). As shown in Table 3, R-index analysis found that the probability of selecting the unseasoned group instead of the Sichuan pepper group and the mixed seasoning group was 100%, indicating that the Sichuan pepper group and the mixed seasoning group had the best adsorption effect on the flavor of fish maw. According to R-value, there was no significant difference between unseasoned group and deionized water group, and no significant difference between Sichuan pepper group and mixed seasoning group. The results obtained by R-index analysis were consistent with those obtained by the rank sum test.

**Table 3:** R-index analysis of flavor adsorption effect of fish maw.

Sample	A	B	C	D	E	F
non-A	0.00	0.48	0.25	0.03	0.00	0.00
non-B	0.52	0.00	0.25	0.02	0.00	0.00
non-C	0.75	0.75	0.00	0.22	0.05	0.04
non-D	0.97	0.98	0.78	0.00	0.13	0.11
non-E	1.00	1.00	0.95	0.87	0.00	0.45
non-F	1.00	1.00	0.96	0.89	0.55	0.00

Note: The value is close to 1, indicating large differences among samples; The value is close to 0.5, indicating random selection among samples. A, B, C, D, E, and F are the same as Table 1.

### 3.2 Analysis of E-nose

**Fig. 1:**
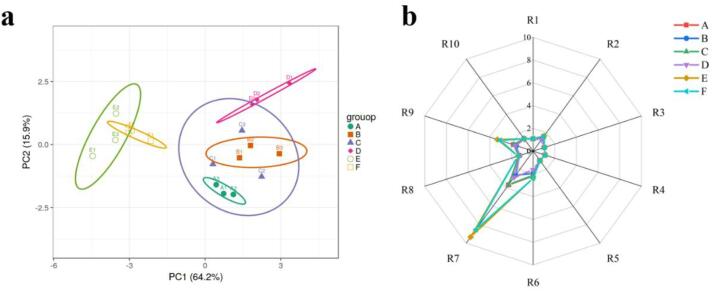
PCA (a) and radar map (b) of E-nose. A, B, C, D, E, and F are the same as Table 1.

**Fig. 2:**
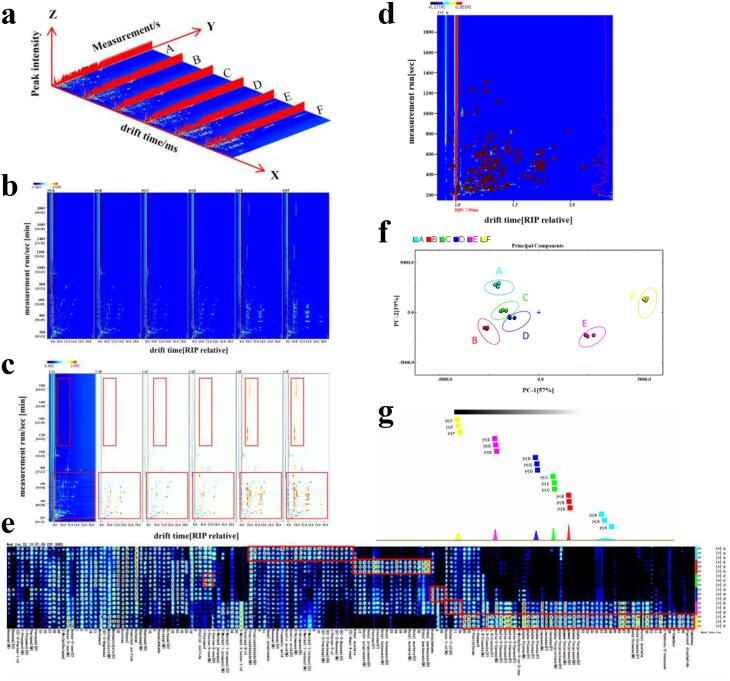
3D-topographic plots (a), 2D-topographic plots (b: vertical view; c: difference view), qualitative analysis (d), gallery plot (fingerprint, e), PCA (f) and analysis of “nearest neighbor” fingerprints (g) of characteristic volatile organic. A, B, C, D, E, and F are the same as Table 1.

E-nose can be effective in obtaining information about volatile compounds in samples (Tiggemann et al., 2017). The E-nose was used to analyze the overall odor of six groups of fish gelatin soaked in different seasonings. Fig. 1a showed the PCA of E-nose. In Fig. 2a, PC1 and PC2 accounted for 64.2% and 15.93% of the total variation, respectively. This finding suggested that the first two PCs could be used in place of the original data. PCA could clearly distinguish the six samples, which were divided into two groups. Samples A, B, C, and D were located on the left side of the score plot, and samples E and F were located on the left side. It was demonstrated that the overall odor of samples A, B, C, and D are similar, but they were different from samples E and F. This might be due to different seasonings used in the soaking process.

The radar map of E-nose was shown in Fig. 1b, and there are some differences in the sensor response values of the six fish maws. The response values of R7 (inorganic sulfides), R9 (organic sulfides and aromatic compounds), and R6 (methyl groups) were more prominent, and the R7 sensor had the highest response value for the fish maw sample, indicating that the R7 sensor could better distinguish the volatile odors of the six groups. In addition, the response values of R7 and R9 sensors in Sichuan pepper group and mixed seasoning group were higher, indicating that the content of inorganic and organic sulfides in these two groups was higher than that in other groups. The response values of the other seven sensors were low, and the odor distribution of fish maw detected by R1 (aromatic components), *R*3 (ammonia and aromatic components), R4 (hydrides), R5 (short chain alkanes), and R10 (long chain alkanes) was almost completely coincident, indicating that the corresponding volatile odor substances were similar. Therefore, the major volatile compounds of the six groups of fish maw were inorganic sulfides, organic sulfides, aromatic compounds, and methyl groups. The results of E-nose were basically consistent with the results of sensory evaluation, but the E-nose was more accurate to distinguish different samples.

### 3.3 Analysis of GC-IMS

#### 3.3.1 HS-GC-IMS spectrum analysis of fish maw

Volatile components of fish maw in six groups (unseasoned group, deionized water group, onion group, ginger group, Sichuan pepper group and mixed seasoning group) were analyzed using FlavourSpec® flavor analyzer. Fig. 2a showed the 3D-topographic plot of HS-GC-IMS spectra of volatile organic compounds in fish maw of different seasoning groups. X-axis represented the retention time, y-axis represented the drift time, and z-axis represented the ion peak. Each point in the spectrum represented a volatile organic compound. The shade of color indicated the level of concentration. White meant low concentration, while red meant high concentration. Compounds might produce 1, 2 or more spots (representing monomers, dimers, or trimers) depending on the concentration and properties of the volatile components (Zhang et al., 2023, Li et al., 2019). As shown in Fig. 2a, the 3D-topographic plot of fish maw in the six groups obtained by LAV analysis software were similar and difficult to distinguish volatile compounds with naked eyes.

As shown in Fig. 2b, the 3D-topographic plot of HS-GC-IMS was projected onto the 2D plane vertical view to obtain the 2D-topographic plot of volatile substances of fish maw in six groups. In order to reflect the differences of volatile components more intuitively between different groups, comparison spectra (Fig. 2c) between samples were drawn with the unseasoned group as the control. As shown in Fig. 2c, the characteristic spectral information of HS-GC-IMS of fish maw soaked in different seasonings was different. This might be related to differences in the characteristic volatile components of different seasonings. And as shown in the red box, some volatile components of fish maw soaked in different seasonings showed significant differences. In particular, the Sichuan pepper and mixed seasoning groups had higher levels of certain volatile compounds than the other four groups, and there was little difference in the number of volatile substances between the two groups. This was consistent with the PCA results of E-nose.

#### 3.3.2 Qualitative analysis of HS-GC-IMS spectra of fish maw

The retention and migration times of characteristic volatile components were compared to calculate the retention index of each volatile component. In addition, *n*-ketone C4-C9 was used as the external standard for qualitative analysis of volatile components by matching GC-IMS database (Han et al., 2021, Song et al., 2021). The marked points shown in Fig. 2d represented the volatile components of qualitative analysis. A total of 95 volatile components (monomers and dimers of some substances) were identified using the software database, including 25 aldehydes, 23 olefins, 19 alcohols, 11 esters, 9 ketones, 3 acids, 2 sulfides, 1 furan, 1 ether and 1 ketoxime. The Monomer and dimer of the same substance had different forms, but had the same CAS number and chemical formula. The numbers in Table 4 corresponded to those in Fig. 2d.

**Table 4:** Identification of volatile components.

No.	Compound	CAS	Formula	MW	RI	Rt [*sec*]	Dt [ms]	Comment
1	Acetic acid	C64197	C_2_H_4_O_2_	60.1	1503.1	984.224	1.05881	Monomer
2	Acetic acid	C64197	C_2_H_4_O_2_	60.1	1501.3	980.94	1.16405	Dimer
3	Benzaldehyde	C100527	C_7_H_6_O	106.1	1528.2	1033.485	1.15544	
4	Nonanal	C124196	C_9_H_18_O	142.2	1402.2	808.954	1.48384	Monomer
5	Nonanal	C124196	C_9_H_18_O	142.2	1402.5	809.468	1.94729	Dimer
6	1-Hexanol	C111273	C_6_H_14_O	102.2	1367.2	755.752	1.33035	
7	6-methylhept-5-en-2-one	C110930	C_8_H_14_O	126.2	1347.3	727.072	1.17886	
8	(Z)-2-Penten-1-ol	C1576950	C_5_H_10_O	86.1	1334.6	709.357	0.94542	
9	Hexyl propionate	C2445763	C_9_H_18_O_2_	158.2	1338.1	714.256	1.43583	
10	octanal	C124130	C_8_H_16_O	128.2	1296.4	658.613	1.41351	Monomer
11	octanal	C124130	C_8_H_16_O	128.2	1294.9	656.629	1.83184	Dimer
12	3-Octanone	C106683	C_8_H_16_O	128.2	1263.4	598.302	1.30671	Monomer
13	3-Octanone	C106683	C_8_H_16_O	128.2	1261.9	595.655	1.71753	Dimer
14	3-Hydroxy-2-butanone	C513860	C_4_H_8_O_2_	88.1	1293.4	653.726	1.07408	
15	(Z)-hept-4-enal	C6728310	C_7_H_12_O	112.2	1251.5	577.641	1.14589	
16	2-pentylfuran	C3777693	C_9_H_14_O	138.2	1237.2	553.75	1.24727	
17	(E)-2-hexenal	C6728263	C_6_H_10_O	98.1	1225.1	534.385	1.17799	Monomer
18	(E)-2-hexenal	C6728263	C_6_H_10_O	98.1	1224.8	533.906	1.51425	Dimer
19	3-Methyl-1-butanol	C123513	C_5_H_12_O	88.1	1212.6	514.979	1.24859	Monomer
20	3-Methyl-1-butanol	C123513	C_5_H_12_O	88.1	1212.1	514.277	1.50034	Dimer
21	heptanal	C111717	C_7_H_14_O	114.2	1188.9	480.202	1.3411	Monomer
22	heptanal	C111717	C_7_H_14_O	114.2	1190.4	482.398	1.70076	Dimer
23	1-Penten-3-ol	C616251	C_5_H_10_O	86.1	1167.9	451.322	0.94148	
24	butan-1-ol	C71363	C_4_H_10_O	74.1	1148.3	426.04	1.18679	Monomer
25	butan-1-ol	C71363	C_4_H_10_O	74.1	1148.3	426.04	1.38873	Dimer
26	(E)-2-pentenal	C1576870	C_5_H_8_O	84.1	1138.0	413.352	1.1004	Monomer
27	(E)-2-pentenal	C1576870	C_5_H_8_O	84.1	1136.6	411.572	1.35844	Dimer
28	diallyl sulfide	C592881	C_6_H_10_S	114.2	1131.6	405.557	1.11722	
29	Hexanal	C66251	C_6_H_12_O	100.2	1088.4	359.323	1.27087	Monomer
30	Hexanal	C66251	C_6_H_12_O	100.2	1089.6	360.308	1.56372	Dimer
31	1-Propanol	C71238	C_3_H_8_O	60.1	1039.7	322.586	1.11157	Monomer
32	1-Propanol	C71238	C_3_H_8_O	60.1	1038.6	321.842	1.25669	Dimer
33	pentanal	C110623	C_5_H_10_O	86.1	982.8	287.046	1.1979	Monomer
34	pentanal	C110623	C_5_H_10_O	86.1	983.3	287.275	1.41903	Dimer
35	Ethanol	C64175	C_2_H_6_O	46.1	921.2	261.479	1.14463	
36	2-methylbutanal	C96173	C_5_H_10_O	86.1	904.8	255.056	1.40415	
37	butanal	C123728	C_4_H_8_O	72.1	872.0	242.666	1.28093	
38	Ethyl acetate	C141786	C_4_H_8_O_2_	88.1	877.1	244.568	1.33788	
39	acetone	C67641	C_3_H_6_O	58.1	813.0	221.929	1.11352	
40	methanol	C67561	CH_4_O	32.0	888.1	248.688	0.98667	
41	dimethyl sulfide	C75183	C_2_H_6_S	62.1	789.4	214.12	0.96235	
42	acetaldehyde	C75070	C_2_H_4_O	44.1	719.4	192.559	0.96489	
43	butyl butanoate	C109217	C_8_H_16_O_2_	144.2	1221.9	529.373	1.34269	Monomer
44	butyl butanoate	C109217	C_8_H_16_O_2_	144.2	1222.1	529.702	1.82285	Dimer
45	styrene	C100425	C_8_H_8_	104.2	1265.8	602.502	1.41319	Polymer
46	styrene	C100425	C_8_H_8_	104.2	1266.1	603.047	1.4403	Polymer
47	butyl propionate	C590012	C_7_H_14_O_2_	130.2	1143.5	420.059	1.28706	Monomer
48	butyl propionate	C590012	C_7_H_14_O_2_	130.2	1142.9	419.334	1.72516	Dimer
49	(E)-2-heptenal	C18829555	C_7_H_12_O	112.2	1329.1	701.819	1.25662	
50	terpinen-4-ol	C562743	C_10_H_18_O	154.3	1638.6	1280.957	1.22831	
51	Limonene	C138863	C_10_H_16_	136.2	1196.7	491.462	1.21644	Monomer
52	Limonene	C138863	C_10_H_16_	136.2	1197.9	493.196	1.30167	Polymer
53	Limonene	C138863	C_10_H_16_	136.2	1197.6	492.701	1.6582	Polymer
54	α-Terpinene	C99865	C_10_H_16_	136.2	1177.2	463.957	1.21332	Monomer
55	α-Terpinene	C99865	C_10_H_16_	136.2	1177.7	464.557	1.72286	Polymer
56	3-Methyl-3-buten-1-ol	C763326	C_5_H_10_O	86.1	1247.7	571.274	1.16778	
57	Dipropyl disulphide	C629196	C_6_H_14_S_2_	150.3	1377.1	770.53	1.26109	
58	α-terpinolene	C586629	C_10_H_16_	136.2	1284.8	637.354	1.21867	Monomer
59	α-terpinolene	C586629	C_10_H_16_	136.2	1285.7	639.015	1.30523	Polymer
60	γ-terpinene	C99854	C_10_H_16_	136.2	1247.3	570.505	1.20944	Monomer
61	γ-terpinene	C99854	C_10_H_16_	136.2	1247.3	570.505	1.70804	Polymer
62	2-Methyl-1-propanol	C78831	C_4_H_10_O	74.1	1100.1	369.632	1.17319	Monomer
63	2-Methyl-1-propanol	C78831	C_4_H_10_O	74.1	1100.4	369.887	1.37203	Dimer
64	butyl acetate	C123864	C_6_H_12_O_2_	116.2	1073.8	347.923	1.24162	Monomer
65	butyl acetate	C123864	C_6_H_12_O_2_	116.2	1074.2	348.178	1.62122	Polymer
66	1-penten-3-one	C1629589	C_5_H_8_O	84.1	1029.2	315.188	1.07913	Monomer
67	1-penten-3-one	C1629589	C_5_H_8_O	84.1	1029.8	315.611	1.31178	Polymer
68	α-Pinene	C80568	C_10_H_16_	136.2	1024.5	311.923	1.21528	Monomer
69	α-Pinene	C80568	C_10_H_16_	136.2	1022.2	310.378	1.30353	Polymer
70	α-Pinene	C80568	C_10_H_16_	136.2	1024.2	311.73	1.66997	Polymer
71	α-Pinene	C80568	C_10_H_16_	136.2	1022.0	310.185	1.737	Polymer
72	Butan-2-one	C78933	C_4_H_8_O	72.1	890.4	249.529	1.06751	Monomer
73	Butan-2-one	C78933	C_4_H_8_O	72.1	894.4	251.07	1.24796	Dimer
74	Propionaldehyde	C123386	C_3_H_6_O	58.1	799.9	217.553	1.0745	Monomer
75	Propionaldehyde	C123386	C_3_H_6_O	58.1	798.7	217.168	1.14445	Dimer
76	β-ocimene	C13877913	C_10_H_16_	136.2	1258.9	590.483	1.21427	Monomer
77	β-ocimene	C13877913	C_10_H_16_	136.2	1258.2	589.166	1.67727	Polymer
78	Δ3-carene	C13466789	C_10_H_16_	136.2	1161.7	443.246	1.2161	Monomer
79	Δ3-carene	C13466789	C_10_H_16_	136.2	1165.7	448.488	1.29243	Polymer
80	Δ3-carene	C13466789	C_10_H_16_	136.2	1162.9	444.801	1.68357	Polymer
81	Δ3-carene	C13466789	C_10_H_16_	136.2	1163.0	444.961	1.72006	Polymer
82	Δ3-carene	C13466789	C_10_H_16_	136.2	1163.4	445.442	2.08639	Polymer
83	1,8-cineole	C470826	C_10_H_18_O	154.3	1206.1	505.227	1.30754	Monomer
84	1,8-cineole	C470826	C_10_H_18_O	154.3	1208.0	508.103	1.72874	Polymer
85	1,8-cineole	C470826	C_10_H_18_O	154.3	1206.8	506.306	2.19551	Polymer
86	ethyl pentanoate	C539822	C_7_H_14_O_2_	130.2	1152.8	431.667	1.26699	Monomer
87	ethyl pentanoate	C539822	C_7_H_14_O_2_	130.2	1151.7	430.265	1.676	Dimer
88	methacrolein	C78853	C_4_H_6_O	70.1	870.2	242.014	1.04991	Monomer
89	methacrolein	C78853	C_4_H_6_O	70.1	876.3	244.273	1.22144	Dimer
90	2-Methyl propanal	C78842	C_4_H_8_O	72.1	808.4	220.374	1.28012	
91	propanoic acid	C79094	C_3_H_6_O_2_	74.1	1608.9	1208.968	1.11813	
92	1-Pentanol	C71410	C_5_H_12_O	88.1	1259.2	591.031	1.51372	
93	d-camphor	C464493	C_10_H_16_O	152.2	1556.1	1091.119	1.3411	
94	Bornyl acetate	C76493	C_12_H_20_O_2_	196.3	1601.0	1190.563	1.22685	
95	Camphene	C79925	C_10_H_16_	136.2	1060.5	337.839	1.21727	

#### 3.3.3 Comparative analysis of HS-GC-IMS fingerprints of fish maw

In order to better compare the differences of volatile components of fish maw soaked in different seasonings. Three parallel tests were conducted on fish maw soaked in different seasonings, and all the peaks of the signals to be identified in the 2D-topographic of HS-GC-IMS were obtained. Finally, the fingerprints of volatile components of fish maw soaked in different seasonings were obtained (Fig. 2e). The rows in Fig. 2e represented samples, and the columns represented the same volatile component in different samples (the darker the red signal, the higher the content). As shown in Fig. 2e, the volatile components of fish maw soaked in different seasonings showed great differences (the red box in Fig. 2e). In the red rectangle area, the contents of *cis*-4-heptenal, *trans*-2-hexenal, 1-pentene-3-ol, *trans*-2-pentenal, 3-methylbutanol, 3-occtanone, acetic acid, propionic acid, nonylaldehyde, ethyl propionate, and propional in the fish maw of the unsoaked group were higher than those in the other five groups. Most of the volatile compounds were fruity, oily and spicy, but there were also some pungent odors such as acetic acid, propionic acid and propionaldehyde, which could produce unpleasant odors. The contents of ethyl valerate, butyl acetate, butyl butyrate, styrene, butyl propionate and ethyl acetate in the fish maw of the deionized water group were higher than those in the other groups, and these volatile compounds mainly exhibited fruity and alcoholic aroma. In contrast, the onion group had higher contents of 1-pentanol and 1-penten-3-one, which were mainly introduced by onion (Wang, Luca, & Edelenbos, 2019). The contents of acetaldehyde and *n*-butanol were higher in ginger group and prickly ash group, respectively. However, the contents of camphene, dipropyl disulfide, d-camphor, 3-hydroxy-2-butanone, bornyl acetate, β-ocimene, α-terpinene, γ-terpinene, α-terpinolene, limonene, α-pinene; 6-methyl-5-hepten-2-one, 3-ene, 1,8-cineole, 1-hexanol, and 2-*n*-pentylfuran in the fish maw of the mixed seasoning group were higher than those in the other groups (Li et al., 2022, Xi et al., 2019). The mixing of seasonings stimulates the formation of a variety of volatile compounds, which have a rich variety of odors. The mixed seasoning group effectively masked some of the unpleasant odors of the fish maw itself, which was consistent with the E-nose results.

#### 3.3.4 Principal component analysis of volatile compounds

In this study, PCA of 95 volatile compounds from fish maw soaked in different seasonings was performed. As shown in Fig. 2f, the contribution of PC1 and PC2 were 57% and 19%, respectively. The cumulative contribution of the two principal components reached 76%. In general, the PCA model could be used as a separation model when the cumulative contribution reaches 60% (Wang et al., 2021). Therefore, PCA could better distinguish fish maw soaked in different seasonings, and there was no overlapping area among the six groups. This indicated that the volatile compounds of fish maw soaked in different seasonings had great differences. It could be seen from the aggregation degree of the groups, the difference between B, C and D was small, indicating that the volatile substances of the deionized water group, onion group and ginger group had little change. However, there were significant differences among E, F and A, indicating that the volatile compounds of Sichuan pepper group and mixed seasoning group varied greatly. This might be because the various volatile aromatic compounds in Sichuan pepper could change the flavor of fish maw. In addition, the “nearest neighbor” fingerprint analysis of fish maw soaked in different seasonings also showed the same results as PCA (Fig. 2g). The order of volatile compounds of fish maw soaked in different seasonings was F > E > D > C > B > A, indicating that volatile compounds in the Sichuan pepper group and mixed seasoning group had greater changes, while those in the deionized water group, onion group and ginger group had smaller changes.

## 4 Conclusion

In this study, sensory evaluation, E-nose, and HS-GC-IMS techniques were used to analyze the flavor changes of fish maw soaked in different seasonings. The volatile compounds of fish maw soaked in different seasonings were mainly organic sulfide and aromatic compounds. The Sichuan pepper group and the mixed seasoning group had better adsorption effect on the flavor of fish maw. The volatile components of fish maw soaked in different seasonings showed great differences. 95 volatile organic compounds were identified by HS-GC-IMS technique. The contents of camphene, dipropyl disulfide, d-camphor, 3-hydroxy-2-butanone, bornyl acetate, β-ocimene, α-terpinene, γ-terpinene, α-terpinolene, limonene, α-pinene, 6-methyl-5-hepten-2-one, 3-ene, 1,8-cineole, 1-hexanol, and 2-*n*-pentylfuran in the fish maw of the mixed seasoning group were higher than those in the other groups. The mixing of seasonings stimulated the formation of a variety of volatile compounds, which had a rich variety of odors and were the major contributors to the flavor of fish maw. This study visualized the volatile flavor characteristics of fish maw soaked with different seasonings, and provided reference for producing high-quality fish maw and improving its flavor quality.

## Declaration of Competing Interest

The authors declare that they have no known competing financial interests or personal relationships that could have appeared to influence the work reported in this paper.

## Data Availability

No data was used for the research described in the article.

## References

[b0005] Chen K.X., Xue L.L., Li Q.Y., Li Y.Y., Mao Y.F., Fan S.W. (2021). Quantitative struc-ture-pungency landscape of sanshool dietary components from Zanthoxylum species. Food Chemistry.

[b0010] Chen M.J., Chen T., Qi X.P., Lu D.L., Chen B. (2020). Analyzing changes of volatile components in dried pork slice by gas chromatography-ion mobility spectroscopy. CyTA - Journal of Food.

[b0015] Chen Y., Zhu K.Y., Zhang Y.Y., Wang S.C., Jin G.W., Jiang P.F. (2021). Analysis of volatile components in different caviar by gas chromatography-ion mobility spectrometry (in Chinese). Food and fermentation industry.

[b0020] Clarke, S. 2002. Trade in dried Asian seafood: characterization, estimation, and implications for conservation. *WCS working paper No. 22.* http://www.wcs.org/science/.

[b0025] Clarke S.C. (2004). Understanding pressures on fishery resources through trade statistics: A pilot study of four products in the Chinese dried seafood market. Fish and Fisheries.

[b0030] Dai, C., Dai, L., Yu, F. J., Li, X. N., Wang, G. X., Chen, J., et al. (2020). Chemical and biological characteristics of hydrolysate of crucian carp swim bladder: focus on preventing ulcerative colitis. *Journal of Functional Foods, 75,* 104256. https://doi.org/10. 1016/j.jff.2020.104256.

[b0035] Feng Q.Q., Hu F., Li P.F. (2012). Analysis of volatile flavor components in Tilapia by SPME-GC-MS (in Chinese). Science and Technology of Food Industry.

[b0045] Han H., Liu C.F., Gao W.C., Li Z.Y., Qin G.W., Qi S.S. (2021). Anthocyanins are converted into anthocyanidins and phenolic acids and effectively absorbed in the jejunum and ileum. Journal of Agricultural and Food Chemistry.

[b0050] Howaili F., Mashreghi M., Shahri N.M., Kompany A., Jalal R. (2020). Development and evaluation of a novel beneficent antimicrobial bioscaffold based on animal waste-fish swim bladder (FSB) doped with silver nanoparticles. Environmental Research.

[b0055] Huang Y., Duan W., Xiao J.F., Liu H., Zhou C.C., Zhang Y.Y. (2020). Characterization of the taste compounds in 20 pungent spices by high-performance liquid chromatography. Journal of Food Measurement and Characterization.

[b0060] Jian J.C., Wu Z.H. (2003). Effects of traditional Chinese medicine on nonspecific immunity and disease resistance of large yellow croaker, Pseudosciaena crocea (Richardson). Aquaculture.

[b0065] Jiang C.Y., Cai W.Q., Shang S., Miao X.Q., Dong X.P., Zhou D.Y. (2022). Comparative analysis of the flavor profile and microbial diversity of high white salmon (*coregonus peled*) caviar at different storage temperatures. LWT-Food Science and Technology.

[b0070] Jiang C.Y., Chen Y., Li S., Shang S., Fu B.S., Wang L.N. (2022). Ready-to-eat fish cake processing methods and the impacts on quality and flavor. Foods.

[b0075] Jin W., Fan X., Jiang C., Liu Y., Zhu K., Miao X. (2023). Characterization of non-volatile and volatile flavor profiles of Coregonus peled meat cooked by different methods. Food Chemistry: X.

[b0080] Jin W., Pei J., Chen X., Geng J., Chen D., Gao R. (2021). Influence of frying methods on quality characteristics and volatile flavor compounds of giant salamander (*Andrias davidianus*) meatballs. Journal of Food Quality.

[b0085] Krüger S., Bergin A., Morlock G.E. (2018). Effect-directed analysis of ginger (*Zingiber officinale*) and its food products, and quantification of bioactive compounds via high-performance thin-layer chromatography and mass spectrometry. Food Chemistry.

[b0090] Li J., Hua J., Dong C. (2020). Real-time fingerprinting of the dynamics of green tea volatiles by ion mobility spectrometry for aroma assessment and discrimination. LWT-Food Science and Technology.

[b0095] Li J.Y., Dadmohammadi Y., Abbaspourrad A. (2022). Flavor components, precursors, formation mechanisms, production and characterization methods: Garlic, onion, and chili pepper flavors. Critical Reviews in Food Science and Nutrition.

[b0100] Li M., Yang R., Zhang H., Wang S., Chen D., Lin S. (2019). Development of a flavor fingerprint by HS-GC-IMS with PCA for volatile compounds of Tricholoma matsutake Singer. Food Chemistry.

[b0105] Li X., Dong Y., Jiang P., Qi L., Lin S. (2022). Identification of changes in volatile compounds in sea cucumber Apostichopus japonicus during seasonings soaking using HS-GC-IMS. LWT-Food Science and Technology.

[b0110] Ma M.M., Mu T.H., Zhou L. (2021). Identification of saprophytic microorganisms and analysis of changes in sensory, physicochemical, and nutritional characteristics of potato and wheat steamed bread during different storage periods. Food Chemistry.

[b0115] Newman J.M. (2004).

[b0120] Peng W.Y., Li P., Ling R.M., Wang Z.Z., Feng X.H., Liu J. (2022). Diversity of volatile compounds in ten varieties of zingiberaceae. Molecules.

[b0125] Sadovy de Mitcheson Y., To A.W., Wong N.W., Kwan H.Y., Bud W.S. (2019). Emerging from the murk: Threats, challenges and opportunities for the global swim bladder trade. Reviews in Fish Biology and Fisheries.

[b0130] Sinthusamran S., Benjakul S. (2015). Effect of drying and frying conditions on physical and chemical characteristics of fish maw from swim bladder of seabass *(Lates calcarife*r). Journal of the Science of Food and Agriculture.

[b0135] Song J.X., Shao Y., Yan Y.M., Li X.H., Peng J., Guo L. (2021). Characterization of volatile profifiles of three colored quinoas based on GC-IMS and PCA. LWT-Food Science and Technology.

[b0140] Tiggemann L., Ballen S.C., Bocalon C.M., Graboski A.M., Manzoli A., Steffens J. (2017). Electronic nose system based on polyaniline films sensor array with different dopants for discrimination of artificial aromas. Innovative Food Science & Emerging Technologies.

[b0145] Wang A., Luca A., Edelenbos M. (2019). Emission of volatile organic compounds from yellow onion (*Allium cepa L.*) bulbs during storage. Journal of Food Science and Technology.

[b0150] Wang F., Gao Y.Q., Wang H.B., Xi B., He X.N., Yang X.L. (2021). Analysis of volatile compounds and flavor fingerprint in Jingyuan lamb of different ages using gas chromatography–ion mobility spectrometry (GC–IMS). Meat Science.

[b0155] Weng Z.B., Sun L., Wang F., Sui X.N., Fang Y., Tang X.Z. (2021). Assessment the flavor of soybean meal hydrolyzed with Alcalase enzyme under different hydrolysis conditions by E-nose, E-tongue and HS-SPME-GC–MS. Food Chemistry: X.

[b0160] Xi J.P., Zhan P., Tian H.L., Wang P. (2019). Effect of spices on the formation of VOCs in roasted mutton based on GC-MS and principal component analysis. Journal of Food Quality.

[b0165] Xu L.R., Wang S.H., Tian A.L., Liu T.R., Benjakul S., Xiao G.S. (2023). Characteristic volatile compounds, fatty acids and minor bioactive components in oils from green plum seed by HS-GC-IMS, GC–MS and HPLC. Food Chemistry: X.

[b0170] Yang Q.Q., Mei X.F., Wang Z.R., Chen X.H., Zhang R., Chen Q.L. (2021). Comprehensive identification of non-volatile bitter-tasting compounds in Zanthoxylum bungeanum Maxim. by untargeted metabolomics combined with sensory-guided fractionation technique. Food Chemistry.

[b0175] Yu Z., Yin L., Qian Y., Yan L. (2009). Effect of Lentinus edodes polysaccharide on oxidative stress, immunity activity and oral ulceration of rats stimulated by phenol. Carbohydrate Polymers.

[b0180] Zhang N., Ayed C., Wang W., Liu Y. (2019). Sensory-guided analysis of key taste-active compounds in pufferfish *(Takifugu obscuru*s). Journal of Agricultural and Food Chemistry.

[b0185] Zhang Y., Tong X.Y., Chen B.J., Wu S.H., Wang X., Zheng Q. (2023). Novel application of HS-GC-IMS for characteristic fingerprints and flavor compound variations in citrus reticulatae pericarpium during storage with different Aspergillus niger fermentation. Food Chemistry: X.

[b0190] Zhao Y.Q., Zeng L., Yang Z.S., Huang F.F., Ding G.F., Wang B. (2016). Anti-fatigue effect by peptide fraction from protein hydrolysate of croceine croaker (*Pseudosciaena crocea*) swim bladder through inhibiting the oxidative reactions including DNA damage. Marine Drugs.

